# Comparison of air displacement plethysmography and octopolar multifrequency bioelectrical impedance analysis in NCAA Division I male lacrosse athletes

**DOI:** 10.7717/peerj.21301

**Published:** 2026-05-27

**Authors:** Jason Curtis Sawyer, Jennifer Elise Hurrell

**Affiliations:** Biological and Biomedical Sciences Department, School of Health and Behavioral Sciences, Bryant University, Smithfield, RI, United States of America

**Keywords:** Fat-free mass, Fat mass, Percent body fat, Agreement analysis, Limits of agreement

## Abstract

**Background:**

Body composition is an important determinant of general health and, in athletes, is often associated with sports performance and injury risk. The purpose of this investigation was to compare body composition measurements gathered through octopolar multifrequency bioelectrical impedance analysis (O-MF-BIA) and air displacement plethysmography (ADP) in Division I lacrosse athletes.

**Materials and Methods:**

Fifty-four male lacrosse players underwent body composition testing using O-MF-BIA and ADP during preseason. Measures of percent body fat (PBF), total fat mass (FM), and total fat-free mass (FFM) were collected using both devices.

**Results:**

There was no statistical difference between O-MF-BIA and ADP for mean values of FFM (*M* = 0.365 kg., *p* = .164), FM (*M* =  − 0.204 kg., *p* = .291), and PBF (*M* = −0.222%., *p* = .302). There was a statistically significant, strong correlation between the O-MF-BIA and ADP for FFM (*r*(52) = .92, *p* < .001), FM (*r*(52) = .75, *p* < .001) and PBF (*r*(52) = .70, *p* < .001). Limits of agreement (LOA) between O-MF-BIA and ADP were determined using Bland-Altman plots for FFM (LOA –4.946 to 5.675), FM (LOA –5.499 to 5.092), and PBF (LOA –6.343 to 5.899).

**Conclusion:**

Body composition measurements obtained by O-MF-BIA and ADP were similar; however, the wide LOA indicates caution should be used when using the devices interchangeably.

## Introduction

### Background

Body composition measurements are often used to quantify health and fitness levels in various human populations (Campa et al., 2021). In athletes, body composition is a determinant of performance and risk of injury (Meir et al., 2001; Domaradzki & Koźlenia, 2022). While body composition research is well established in major revenue sports like football and soccer, male lacrosse players remain under-represented in the literature (Collins et al., 2014). This research gap is particularly notable given the unique physiological demands of men’s lacrosse. Unlike football, where maximal size is often advantageous, or soccer, where high levels of lean mass are prioritized, lacrosse requires a unique blend of power for collision play and significant aerobic capacity for continuous field movement (Akiyama, Sasaki & Mashiko, 2019). Despite rapid growth in men’s lacrosse participation at the collegiate level, the lack of body composition research limits practitioners’ ability to optimize training and nutritional interventions for performance enhancement and injury risk reduction (Ripley, Wenham & Collier, 2025). Establishing validated measurement methods for body composition in male lacrosse players is therefore essential to address this knowledge gap.

Body composition can be analyzed at the tissue level by categorizing body mass into fat-free mass (FFM) and fat mass (FM). Fat-free mass is comprised of skeletal muscle and other lean soft tissue (Campa et al., 2021). Fat mass includes visceral adipose tissue and subcutaneous adipose tissue (SAT) (Shuster et al., 2012). There are several field-based and laboratory techniques that can be used to estimate body composition; however, each has inherent limitations relating to either the methodology or assumptions required for a valid test (Fosbøl & Zerahn, 2015). Using a height:weight index, such as the body mass index (BMI) involves minimal cost, time, and skill; however, BMI is unable to discriminate between mass originating from muscle *versus* adipose tissue (Bushman, 2022). Therefore, BMI is not always a valid measure of body composition because it can fall in the normal range when individuals have a combination of high fat mass and low lean mass, and it can overestimate percent body fat in individuals with a higher degree of muscle mass (Frankenfield et al., 2001). Multiple researchers (Houska et al., 2018; Garrido-Chamorro et al., 2009) determined that BMI is an unreliable indicator of body composition in athletic populations, including elite athletes.

Previous research supports that hydrostatic weighing (HW), air displacement plethysmography (ADP), and dual-energy X-ray absorptiometry (DXA) are commonly used as criterion measurements for body composition (Campa et al., 2021; Fosbøl & Zerahn, 2015; Orsso et al., 2020; Von Hurst et al., 2016). In a summary of seventeen studies that examined percent body fat (PBF) measurements obtained using ADP and HW, there was less than a one percent difference between the two methods (Fields, Goran & McCrory, 2002). Another systematic review of 66 studies using a variety of techniques to measure body composition, ADP, and DXA were among those having the highest agreement with reference standards (Orsso et al., 2020).

While evidence suggests the validity of the established criterion methods for measuring body composition, access to these devices may be limited. Bioelectrical impedance analysis (BIA) is becoming more commonplace due to its affordability, minimal invasiveness, portability, and ease of use (Ceniccola et al., 2019). BIA measures resistance to the flow of a low-intensity electric current through the body to indirectly estimate body composition (Ceniccola et al., 2019). Conductivity is positively correlated with higher levels of total body water (TBW) because current flows more readily through fluid rich in electrolytes (*i.e.,* FFM such as skeletal muscle) (Fosbøl & Zerahn, 2015). However, FM is a poor electrical conductor. Utilizing BIA conductance measures, proprietary algorithms are used to determine body composition measurements such as absolute measures of FFM, FM, and PBF.

Studies have assessed how BIA compares to established criterion measures when measuring body composition variables such as FFM, FM, and PBF. Despite varying levels of correlation and statistical significance, when comparing BIA to DXA, several studies have found the differences in mean PBF were less than 4% in DI collegiate athletes (Brewer et al., 2019; Raymond, Bosch & Dengel, 2017), collegiate athletes of varying divisions (Esco et al., 2015), national rugby players (Delaney et al., 2016), and adults with a wide range of BMIs (Von Hurst et al., 2016), with BIA underestimating PBF in all cases. Other researchers reported that BIA and ADP have yielded similar results for PBF, with differences under 4% in children, adults, athletes, and clinical populations (Von Hurst et al., 2016; Mahaffey et al., 2023; Benton et al., 2011; Foucart et al., 2017; Wong Vega & Srivaths, 2017; Ferri-Morales et al., 2018). Furthermore, Schlösser et al. (2022) compared the accuracy of DXA, ADP, BIA, and anthropometry for assessing body composition in elite soccer referees and concluded that ADP followed by BIA were the most accurate methods for evaluating PBF. In cases where statistically significant differences between BIA and the criterion measure were present, the authors concluded that differences up to 6% were clinically acceptable (Mahaffey et al., 2023).

There are a variety of BIA devices on the market, such as single frequency (SF-BIA) *versus* multifrequency (MF-BIA), which have the benefit of utilizing multiple frequencies to more accurately assess body composition (Gába et al., 2015). According to Silva et al. (2019), SF-BIA and MF-BIA cannot be used interchangeably due to a lack of agreement between devices. In addition, the number of contact points on the body can vary with bipolar devices that make two points of contact (*i.e.,* hand to hand, foot to foot, hand to foot, *etc*.), quad polar that makes four points of contact, *versus* octopolar devices that encompass simultaneous contact with two electrodes on each foot, and two electrodes on each hand (Jaffrin & Morel, 2009). Because of the varied options for frequency and points of body contact, making global conclusions about the accuracy of BIA technology is difficult (Dzator et al., 2023). Instead, conclusions regarding the accuracy of BIA are only valid for the particular BIA device studied.

### Purpose

The primary purpose of this study was to compare body composition measurements gathered through octopolar multifrequency bioelectrical impedance analysis (O-MF-BIA) and ADP in Division I male lacrosse athletes. To our knowledge, there are no known studies comparing these modalities in this athletic population. The secondary purpose was to determine the correlation between BMI and PBF, as measured by ADP *vs.* BIA.

## Materials & Methods

### Design

A cross-sectional approach was utilized to measure body composition, with all measurements completed in randomized order during a single testing session. Prior to testing, subjects were asked to complete an overnight fast, refrain from any physical activity for three hours before testing and abstain from alcohol ingestion for 24 h. Subjects reported to the laboratory between 07:30 and 09:30 am Eastern Standard Time (EST). Upon arrival, subjects completed an informed consent and medical history questionnaire (MHQ). Subjects then emptied their bladder prior to testing. The height of subjects was measured using a standard stadiometer, measured to the nearest cm. Subjects then underwent body composition testing using O-MF-BIA and ADP technology.

This study was conducted according to the guidelines outlined in the Declaration of Helsinki, and all procedures involving human subjects were approved by the Bryant University Institutional Review Board [IRB Proposal #2023-1108]. Written informed consent was obtained from all subjects.

### Subjects

Fifty-four (*n* = 54) National Collegiate Athletic Association (NCAA) Division I male lacrosse players were recruited for this study. Inclusion criteria included subjects who were participating in preseason training and were at least 18 years of age at the time of testing. Subjects were excluded from the study if they had a pacemaker or were claustrophobic; these are contraindications for BIA and ADP, respectively. Refer to Table 1 for subject demographic information.

### Methodology

#### Air displacement plethysmography

Body density was measured *via* ADP technology using a COSMED Bod Pod (Life Measurement Instruments, Inc., Concord, CA, USA). The Siri prediction equation was then applied to determine FFM and FM. Prior to testing, the equipment was calibrated following the manufacturer’s guidelines. To minimize the amount of air trapped by clothing and hair, subjects wore tight-fitting Lycra shorts and a swim cap per the manufacturer’s recommendations. All jewelry was removed prior to testing. Body weight was determined using a COSMED calibrated digital scale (Life Measurement Instruments, Inc., Concord, CA, USA). Subjects were instructed to sit in the Bod Pod quietly and to minimize movement during testing. Two measures of body volume were completed. If there was a difference of more than 150 mL between measurements, a third body volume measurement was taken. When three measurements were required, the final body volume was determined by averaging the two closest values. Predicted lung volumes were utilized during ADP testing. Previous researchers (Foote et al., 2022; Wellens, 1996) determined that there are no significant differences between measured and predicted lung volumes in healthy adults.

#### Octopolar multifrequency bioelectrical impedance analysis

O-MF-BIA measurements were obtained using an Inbody 570 (InBody Co., Ltd., Seoul, Korea). Subjects stood barefoot in the upright position with the heels and forefeet placed on the posterior and anterior sections of the footpads, respectively. Handles were gripped with the thumbs, and fingers placed on the electrodes, and the arms were held in slight abduction. During measurement, subjects were asked to refrain from talking and to minimize movement.

**Table 1:** Mean age, height, BMI, body mass and body composition measurements determined by ADP and BIA.

Value	Mean ± SD	
Age (years)	20.84 ± 1.54	
Height (cm)	181.53 ± 6.78	
BMI weight/height^2^	25.98 ± 1.86	
	ADP	BIA
Body Mass (kg)	85.54 ± 6.93	85.70 ± 6.95
FFM (kg)^1^	75.207 ± 6.25	75.571 ± 6.80
FM (kg)^2^	10.336 ± 3.92	10.132 ± 3.76
PBF (%)^3^	12.007 ± 4.02	11.785 ± 4.04

**Notes.**

1 FFM, fat-free mass.

2 FM, fat mass.

3 PBF, percent body fat.

#### BMI

BMI was calculated using weight measurements collected by the InBody 570 and height measurements collected by the stadiometer, using the formula BMI = weight/height^2^ (Meir et al., 2001).

### Statistical analysis

All statistical analyses were completed using SPSS for Windows, version 28 (SPSS, Inc., Chicago, IL.) Paired-sample t-tests were utilized to determine mean differences between O-MF-BIA and ADP for FFM, FM, and PBF. To decrease the chance of a Type I error, a Bonferroni-adjusted *p* value was used to test for significant differences. The adjusted *p* value used for this research was 0.0167. Cohen’s *d* statistic was used to determine the magnitude of effect size. A Pearson’s Correlation was calculated to determine the association between O-MF-BIA and ADP for FFM, FM, and PBF. The Bland-Altman method was used to determine the 95% limits of agreement between methods. The mean difference was calculated as the mean difference between O-MF-BIA and ADP methods, and 95% limits of agreement (LOA) were calculated using the mean difference ± 1.96 (SD). Proportional bias was assessed by calculating Pearson’s correlation coefficient between the difference scores and the mean of the two methods. An alpha level of 0.05 was used to assess significant proportional bias. Post hoc power analyses were conducted using G*Power, version 3.1.9.7 (Faul et al., 2007) to determine the power for detecting the difference between O-MF-BIA and ADP measurements.

## Results

There was no statistical difference between O-MF-BIA and ADP for FFM (*p* = .16), FM (*p* = .29), and PBF (*p* = .30). Measurements of PBF were 0.222% lower when assessed using BIA as compared to ADP. A body composition comparison is presented in Table 2. The post hoc power analysis indicated low achieved power for FFM (1 − *β* = 0.077), FM (1 − *β* = 0.033), and PBF (1 − *β* = 0.032). The calculated effect sizes were trivial for FFM (*d* = 0.135), FM (*d* = 0.075), and PBF (*d* = 0.071), indicating that differences between devices were negligible and not clinically meaningful.

**Table 2:** Body composition comparison between O-MF-BIA and ADP for body composition variables.

			95% Confidence Intervals	
Value	Mean ± SD	*p*	Lower	Upper
FFM (kg)	0.365 ± 5.960	.164	−0.375	1.105
FM (kg)	−0.204 ± 5.940	.291	−0.941	0.533
PBF (%)	−0.222 ± 3.123	.302	−0.489	0.286

**Notes.**

Abbreviations FFM fat-free mass FM fat mass PBF percent body fat

There was a statistically significant, strong correlation between the O-MF-BIA and ADP for FFM (*r*(52) = .92, *p* < .001), FM (*r*(52) = .75, *p* < .001) and PBF (*r*(52) = .70, *p* < .001). The explained variance between the two devices for FFM, FM, and PBF was 84%, 56%, and 49%, respectively.

There were no violations of assumptions for t-tests or correlation analysis as evidenced by: (1) there were no outliers in the difference scores for FFM, FM, PBF, as assessed by inspection of a boxplot, (2) the difference scores for the O-MF-BIA and ADP devices were normally distributed as assessed by Shapiro–Wilk’s test for FFM (*p* = .78), FM (*p* = .75), and PBF (*p* = .26), and (3) there was a linear relationship between O-MF-BIA and ADP for FFM, FM and PBF as visualized by inspection of scatterplots.

**Figure 1:**
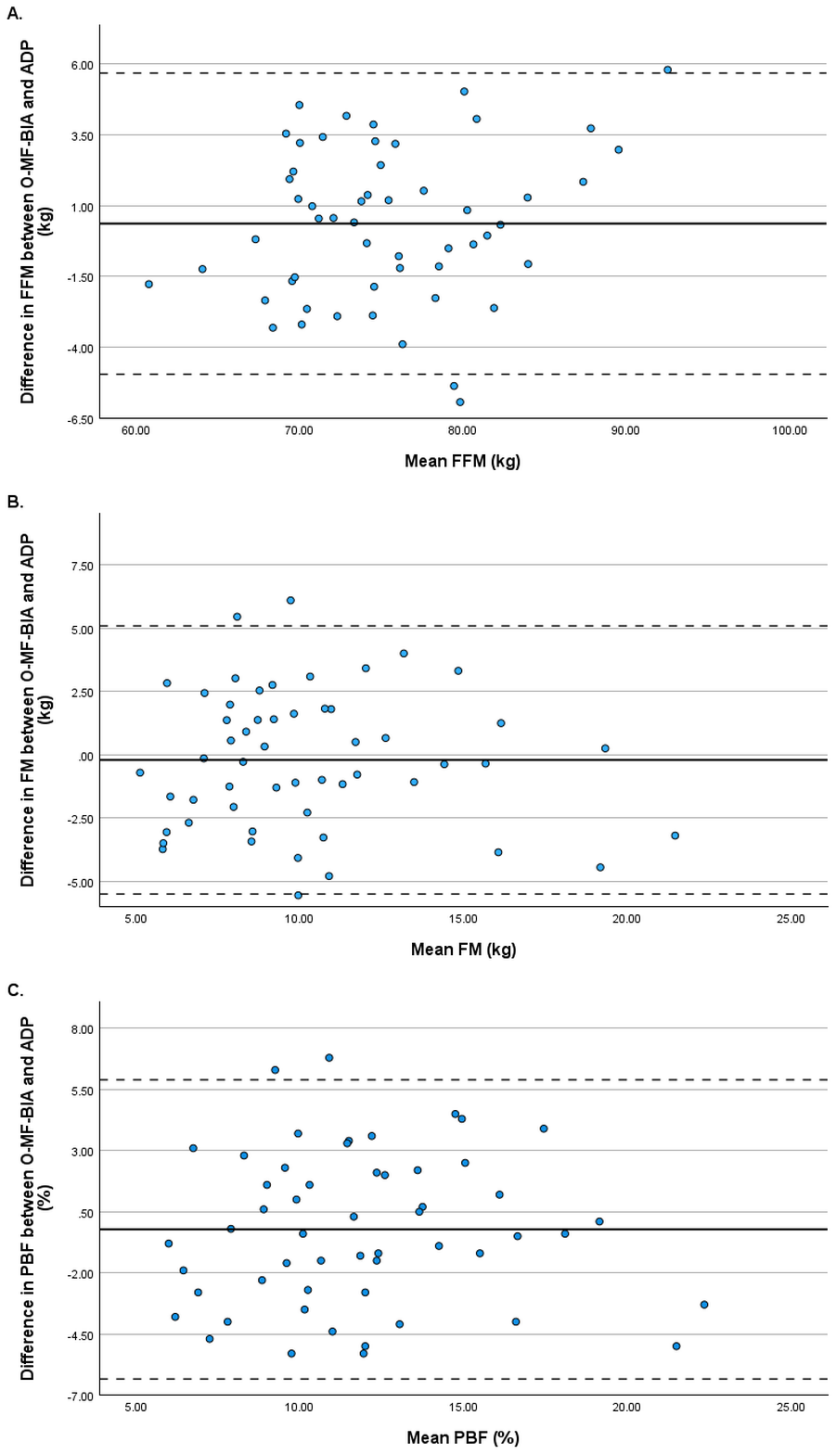
Bland-Altman Plots for (A) Fat-Free Mass, (B) Fat Mass, and (C) Percent Body Fat. Abbreviations: FFM, fat-free mass; FM, fat mass; PBF, percent body fat.

Limits of agreement between O-MF-BIA and ADP were determined using Bland-Altman plots for FFM (LOA −4.946 to 5.675), FM (LOA −5.499 to 5.092), and PBF (LOA −6.343 to 5.899). Bland-Altman plots for measures of body composition are provided in Fig. 1. The trends between the difference and the mean for O-MF-BIA and ADP were not significant for FFM (*r* = .03, *p* = .13), FM (*r* = .06, *p* = .64), and PBF (*r* = .01, *p* = .96).

There was a statistically significant, strong correlation between BMI and PBF as measured by BIA (*r*(52) = .57, *p* < .001), and a statistically significant, moderate correlation between BMI and PBF as measured by ADP (*r*(52) = .41, *p* = .002).

## Discussion

The primary finding of this research study is that there were no significant differences between O-MF-BIA and ADP for measuring mean values of FFM, FM, and PBF in the male Division I lacrosse athletes studied. The mean difference in PBF in this study was 0.222% and based on prior research (Mahaffey et al., 2023), differences in PBF between methods of up to 5–6% are generally considered acceptable for practical use. Furthermore, there were strong correlations between O-MF-BIA and ADP for measures of FFM, FM, and PBF. Despite a lack of significant mean differences, the LOAs were wide between devices. A secondary finding of this research study is that BMI was strongly to moderately associated with PBF, as measured by BIA and ADP, respectively.

### Comparing ADP *versus* Single, Bipolar, and Quadripolar BIA

Previous research comparing PBF measurements taken with ADP and BIA devices that were single frequency, bipolar, or quadripolar in nature, levels of agreement have varied. Mahaffey et al. (2023) and Wong Vega & Srivaths (2017) both found that BIA underestimated PBF in populations of obese children. However, Wong Vega & Srivaths (2017) concurrently, found good agreement between ADP and BIA in normal weight children. Other authors reported that BIA underestimates PBF in 12–14-year-old male athletes, especially those with higher adiposity, Ferri-Morales et al. (2018) and in healthy females aged 40–55 (Benton et al., 2011).

### Comparing ADP *versus* Octopolar BIA

Studies comparing PBF measured by ADP and O-MF-BIA have reported varying levels of agreement. Foucart et al. (2017) examined the agreement between ADP and O-MF-BIA in children aged 7–13 years and determined a wide limit of agreement between devices. Additionally, O-MF-BIA tended to overestimate PBF in children with low PBF and underestimate PBF in children with high PBF. Similarly, Von Hurst et al. (2016) found that compared to ADP, O-MF-BIA underestimated PBF in the presence of higher adiposity, and overestimated PBF in the presence of lower adiposity levels in healthy adults. Houska et al. (2018) determined that O-MF-BIA overestimated PBF in lean females. The PBF values of the subjects in our study fell within the normal range, which may partially explain the lack of significant differences between the devices.

### Comparing Octopolar BIA *versus* DXA

Several studies examined PBF measurements taken with O-MF-BIA compared to the criterion assessment method of DXA. Researchers reported that O-MF-BIA underestimated PBF in DI and other collegiate athletes (Brewer et al., 2019; Raymond, Bosch & Dengel, 2017; Esco et al., 2015) and in healthy adults (Sillanpää et al., 2014; Völgyi et al., 2008). In contrast, Ling et al. (2011) found excellent agreement between O-MF-BIA and DXA for PFB measurements in those with normal levels of body fat and those with excess adiposity. Similarly, Shafer et al. (2009) compared O-MF-BIA to DXA and found less than a 2% difference between methods in individuals with BMI in the normal and overweight ranges. Based on their interpretation that “the biological significance of these errors is small” they concluded that BIA is a valid measurement method for this population. Esco et al. (2015) examined the agreement between O-MF-BIA and DXA in Division I Collegiate Baseball players. While the researchers found significant differences between the two methods for FM and PBF, no differences existed between FFM measures in the arms, trunk, legs, and whole body.

### Comparing BIA based on frequency and contact electrodes

Demura, Sato & Kitabayashi (2004) compared various BIA devices, including single frequency with four contact electrodes, single frequency with eight contact electrodes, and multifrequency with eight contact electrodes, with DXA and HW. The researchers determined that O-MF-BIA had the highest agreement and lowest standard error of estimation (SEE) with criterion measures compared to other BIA devices. The InBody 570 used in this study is an O-MF-BIA device, and therefore, the accuracy may be improved compared to other BIA devices. Similar to Ling et al. (2011) and Shafer et al. (2009), the current study found that the InBody O-MF-BIA device produced PBF measurements comparable to those obtained from the criterion method.

### Correlation of BIA measurements to criterion measurement

There was concordance between the body composition measurements obtained through O-MF-BIA and ADP in the current study, and thus there was a strong correlation between the two sets of values. However, it should be noted that even when significant differences are present between BIA and criterion measures, body composition measurements obtained through BIA tend to be highly correlated to those obtained using criterion measurement techniques (Von Hurst et al., 2016; Esco et al., 2015; Benton et al., 2011; Company & Ball, 2010).

### BMI correlation with BIA derived body composition measurements

Similar to the results of the current study, previous researchers (Garrido-Chamorro et al., 2009; Suchanek et al., 2012; Ranasinghe et al., 2013) reported moderate to strong correlations between body composition measures and BMI. Suchanek et al. (2012) determined a high correlation between BMI and quad polar-MF-BIA measures of PBF in adult females. Furthermore, in a study of adults aged 18–83 years, Ranasinghe et al. (2013) found a strong correlation between BMI and PBF measured by octopolar single frequency BIA. However, the strength of the correlation between PBF and BMI was significantly influenced by age and gender (Ranasinghe et al., 2013). In the current study, the homogenous nature of the subjects, who were similar in age and all identified as male, may explain the moderate and strong correlations observed between BMI and PBF measured through ADP and O-MF-BIA.

In the current study, the mean BMI of the subjects was 25.9 kg/m^2^, which is a classification of “overweight” (WHO, 1995). However, the mean PBF for O-MF-BIA and ADP were 11.79% and 12.00%, respectively. The American College of Sports Medicine (ACSM) ranks these scores in the “good” category for PBF (ACSM, 2021). In a study of athletes, Kruschitz et al. (2013), determined that BMI categorized athletes as overfat despite having normal PBF. The authors attributed this difference to an increase in skeletal muscle mass in athletes, which BMI lacks the ability to discern between tissue types. Heymsfield et al. (2009) examined the correlation between BMI and body composition measured by MRI. The researchers determined that skeletal muscle was a significant contributor to BMI classification. Division I athletes typically engage in strength training, particularly during the off-season and pre-season, which may systematically skew BMI values for this population (Heymsfield et al., 2009).

### Limits of agreement

Despite a lack of significant differences between ADP and O-MF-BIA for body composition measures in the current study, there were wide LOAs between devices in this study. The range of LOAs for FFM, FM, and PBF were 10.621 kg, 10.591 kg, and 12.242%, respectively. Similarly, Ferri-Morales et al. (2018) determined wide LOAs between BIA and ADP in youth swimmers, footballers, and cyclists. Moreover, wide LOA were reported between O-MF-BIA and DXA for overall FFM, FM, and PBF in collegiate football players (Raymond, Bosch & Dengel, 2017). The wide LOAs have important practical implications for athlete monitoring. In the strength and conditioning setting, coaches and practitioners often track small changes in body composition as evidence of the effectiveness of a training program and nutritional intervention. The wide LOAs in the current study may exceed typical thresholds, meaning that apparent changes in body composition may reflect measurement error rather than true physiological adaptations. Therefore, while O-MF-BIA and ADP produce similar group-level estimates, the devices should not be used interchangeably for individual athlete monitoring.

### Limitations

Hydration status was not directly measured, which represents a methodological constraint given that O-MF-BIA measurements are sensitive to fluctuations in total body water (Brewer et al., 2019). While subjects were instructed to follow a rigorous pre-testing protocol, individual variations in hydration status could have influenced the agreement between methods. The current investigation was meant to imitate athlete testing conditions in a strength and conditioning facility. Determining hydration status is cumbersome and is not typically feasible in the strength and conditioning setting. Similarly, food intake prior to testing was not directly monitored; however, subjects were instructed to arrive to the laboratory in a fasted state. The use of predicted rather than measured lung volumes represents another methodological constraint. Previous researchers (Foote et al., 2022; Wellens, 1996) reported no significant differences between predicted and measured lung volumes in healthy adults; individual differences in lung capacity could have introduced small errors in body volume calculations. Because the current study included only male lacrosse athletes, the findings should not be generalized to other sexes or sports. Future research should examine the validity of O-MF-BIA for monitoring longitudinal body composition changes and expand to include other sports.

## Conclusions

In Division I male lacrosse athletes, mean body composition measures derived from O-MF-BIA and ADP were similar, with moderate to strong correlations. However, the wide limits of agreement suggest that the two devices are not interchangeable. Caution is therefore warranted when comparing results derived from each device. O-MF-BIA may be a practical alternative to ADP in sports settings; however, consistent use of the same device is recommended for long-term monitoring of athletes. While the devices should not be used interchangeably for individual assessments, either method may be suitable for generating population-level estimates of body composition.

##  Supplemental Information

10.7717/peerj.21301/supp-1 Supplemental Information 1 Raw Data

10.7717/peerj.21301/supp-2 Supplemental Information 2 STROBE checklist

## Additional Information and Declarations

### Data Availability

The following information was supplied regarding data availability: The raw measurements are available in the Supplemental File.
