# Supplementary material for: Comparison of air displacement plethysmography and octopolar multifrequency bioelectrical impedance analysis in NCAA Division I male lacrosse athletes
Source: PeerJ. 2026 May 27;14:e21301. doi: 10.7717/peerj.21301 (PMC13221984; doi:10.7717/peerj.21301)
Supplement: Supplemental Information 2 [file peerj-14-21301-s002.docx]

STROBE Statement—checklist of items that should be included in reports of observational studies

|  | Item No. | Recommendation | Page  No. | Relevant text from manuscript |  |  |  |
| --- | --- | --- | --- | --- | --- | --- | --- |
| **Title and abstract** | 1 | (*a*) Indicate the study’s design with a commonly used term in the title or the abstract | p.1 (Abstract) | Cross-sectional approach stated in abstract: "A cross-sectional approach was utilized to measure body composition, with all measurements completed in a single session." |  |  |  |
|  |  | (*b*) Provide in the abstract an informative and balanced summary of what was done and what was found | p.1 | Abstract includes background, methods, results, and conclusions. Background describes body composition importance in athletes. Methods describe 54 male lacrosse players tested with O-MF-BIA and ADP. Results report no statistical differences between methods with strong correlations. Conclusion notes similar measurements but cautions about wide limits of agreement. |  |  |  |
| Introduction | | | |  |  | pp.2–4 | Scientific background and rationale described. |
| Background/rationale | 2 | Explain the scientific background and rationale for the investigation being reported | pp.1-2 | Body composition background is explained, including its importance in athletes for performance and injury risk. Different measurement methods are discussed, including limitations of BMI, validity of criterion methods (HW, ADP, DXA), and the growing use of BIA. Specifics regarding ADP and various BIA devices (single vs. multifrequency, bipolar vs. octopolar) are provided. Rationale established that conclusions about BIA accuracy are device-specific. |  |  |  |
| Objectives | 3 | State specific objectives, including any prespecified hypotheses | p.2 | Primary objective: "to compare body composition measurements gathered through octopolar multifrequency bioelectrical impedance analysis (O-MF-BIA) and ADP in Division I male lacrosse athletes." Secondary objective: "to determine the correlation between BMI and PBF, as measured by ADP vs. BIA." |  |  |  |
| Methods | | | |  |  | p.5 | Laboratory, preseason testing, morning sessions. |
| Study design | 4 | Present key elements of study design early in the paper | pp.2-3 | The cross-sectional design is clearly stated: "A cross-sectional approach was utilized to measure body composition, with all measurements completed in a single session." Testing procedures are explained in detail including pre-testing requirements (overnight fast, 3-hour activity restriction, 24-hour alcohol abstinence), randomized order of testing, and specific methodology for each device. |  |  |  |
| Setting | 5 | Describe the setting, locations, and relevant dates, including periods of recruitment, exposure, follow-up, and data collection | pp.2-3 | Testing was conducted at Bryant University during the preseason period. Subjects reported to the laboratory between 07:30 and 09:30 am EST. All measurements were completed in a single session. The study was approved by Bryant University Institutional Review Board [IRB Proposal #2023-1108] and conducted according to the Declaration of Helsinki guidelines. |  |  |  |
| Participants | 6 | (*a*) *Cohort study*—Give the eligibility criteria, and the sources and methods of selection of participants. Describe methods of follow-up  *Case-control study*—Give the eligibility criteria, and the sources and methods of case ascertainment and control selection. Give the rationale for the choice of cases and controls  *Cross-sectional study*—Give the eligibility criteria, and the sources and methods of selection of participants | p.3 | Fifty-four (n = 54) NCAA Division I male lacrosse players participating in preseason training were recruited. Inclusion criteria: participating in preseason training and at least 18 years of age at the time of testing. Exclusion criteria: presence of pacemaker (contraindication for BIA) or claustrophobia (contraindication for ADP). All subjects provided written informed consent and completed a medical history questionnaire. Demographic information is provided in Table 1. |  |  |  |
|  |  | (*b*) *Cohort study*—For matched studies, give matching criteria and number of exposed and unexposed  *Case-control study*—For matched studies, give matching criteria and the number of controls per case | N/A |  |  |  |  |
| Variables | 7 | Clearly define all outcomes, exposures, predictors, potential confounders, and effect modifiers. Give diagnostic criteria, if applicable | pp.2-3 | Primary outcome variables clearly defined: percent body fat (PBF), total fat mass (FM), and total fat-free mass (FFM) measured by both O-MF-BIA and ADP. Body density measured via ADP using COSMED Bod Pod with Siri prediction equation applied to determine FFM and FM. O-MF-BIA measurements obtained using Inbody 570. Secondary outcome: BMI calculated using weight/height² formula. No confounders were adjusted for in this comparison study. |  |  |  |
| Data sources/ measurement | 8* | For each variable of interest, give sources of data and details of methods of assessment (measurement). Describe comparability of assessment methods if there is more than one group | p.3 | ADP: Body density measured via COSMED Bod Pod (Life Measurement Instruments, Inc., Concord, CA, USA). Equipment calibrated per manufacturer guidelines. Subjects wore tight-fitting Lycra shorts and swim cap, removed all jewelry. Two body volume measures completed (third if difference >150 mL). Predicted lung volumes utilized. O-MF-BIA: Measurements obtained using Inbody 570 (InBody Co., Ltd., Seoul, Korea). Subjects stood barefoot with heels/forefeet on footpads, gripped handles with thumbs/fingers on electrodes, arms in slight abduction. Height: measured using standard stadiometer to nearest cm. Both methods assessed same variables (FFM, FM, PBF) for direct comparison. |  |  |  |
| Bias | 9 | Describe any efforts to address potential sources of bias | pp.2-3 | Multiple efforts to address bias: (1) Subjects completed overnight fast, refrained from physical activity for 3 hours, and abstained from alcohol for 24 hours before testing to control for hydration and metabolic state; (2) Testing conducted in morning (07:30-09:30 am EST) to standardize circadian effects; (3) Randomized order of O-MF-BIA and ADP testing to control for order effects; (4) Equipment calibrated following manufacturer guidelines; (5) Standardized clothing (Lycra shorts, swim cap for ADP) to minimize air trapping; (6) Predicted lung volumes used for ADP as research shows no significant differences from measured volumes in healthy adults; (7) Statistical assumptions verified (outliers, normality, linearity). |  |  |  |
| Study size | 10 | Explain how the study size was arrived at | p.3 | Fifty-four (n = 54) NCAA Division I male lacrosse players were recruited for this study. Sample size represents the available population of Division I male lacrosse players participating in preseason training who met inclusion criteria (at least 18 years of age) and did not meet exclusion criteria (pacemaker or claustrophobia). |  |  |  |

Continued on next page

| Quantitative variables | 11 | Explain how quantitative variables were handled in the analyses. If applicable, describe which groupings were chosen and why | pp.3-4 | All variables (FFM, FM, PBF) were treated as continuous quantitative variables. No groupings or categorizations were applied. The statistical analysis section describes SPSS version 28 software used for analysis. Bonferroni-adjusted p value of 0.0167 was used to decrease Type I error risk. Cohen's d statistic calculated for effect size magnitude. Pearson's Correlation calculated for associations. Bland-Altman method used to determine 95% limits of agreement with mean difference calculated as O-MF-BIA minus ADP, and 95% LOA calculated using mean difference ± 1.96(SD). Alpha level of 0.05 used to assess significant trends in Bland-Altman plots. |
| --- | --- | --- | --- | --- |
| Statistical methods | 12 | (*a*) Describe all statistical methods, including those used to control for confounding | pp.3-4 | Paired-sample t-tests utilized to determine mean differences between O-MF-BIA and ADP for FFM, FM, and PBF. Bonferroni-adjusted p value of 0.0167 used to control for Type I error. Cohen's d statistic calculated for effect size. Pearson's Correlation calculated for associations between methods and between BMI and PBF. Bland-Altman method used for 95% limits of agreement. Statistical assumptions verified: no outliers (boxplot inspection), normal distribution (Shapiro-Wilk test), and linear relationships (scatterplot inspection). No confounders were controlled for as this was a method comparison study. |
|  |  | (*b*) Describe any methods used to examine subgroups and interactions | N/A | No subgroup analyses or interaction terms were examined. |
|  |  | (*c*) Explain how missing data were addressed | N/A | No missing data reported. All 54 participants completed both measurement methods. |
|  |  | (*d*) *Cohort study*—If applicable, explain how loss to follow-up was addressed  *Case-control study*—If applicable, explain how matching of cases and controls was addressed  *Cross-sectional study*—If applicable, describe analytical methods taking account of sampling strategy | N/A | Convenience sample of available Division I male lacrosse players. No complex sampling strategy requiring analytical adjustment. |
|  |  | (*e*) Describe any sensitivity analyses | N/A | No sensitivity analyses were conducted. |
| Results | | | | |
| Participants | 13* | (a) Report numbers of individuals at each stage of study—eg numbers potentially eligible, examined for eligibility, confirmed eligible, included in the study, completing follow-up, and analysed | p.3 | Fifty-four (n = 54) NCAA Division I male lacrosse players were recruited and all completed the study. All 54 participants were included in the analysis. No participants were excluded after enrollment. Statistical assumption checks confirmed no outliers in difference scores as assessed by boxplot inspection, normal distribution of difference scores (Shapiro-Wilk test: FFM p=.78, FM p=.75, PBF p=.26), and linear relationships between methods. |
|  |  | (b) Give reasons for non-participation at each stage | N/A | Not reported. All enrolled participants completed the study. |
|  |  | (c) Consider use of a flow diagram | N/A | No flow diagram included. Simple study design with single testing session and no attrition. |
| Descriptive data | 14* | (a) Give characteristics of study participants (eg demographic, clinical, social) and information on exposures and potential confounders | p.3, Table 1 | Participant characteristics provided in Table 1: n=54 NCAA Division I male lacrosse players. All subjects were at least 18 years of age and participating in preseason training. Mean BMI was 25.9 kg/m² (classified as "overweight" by WHO standards). Mean PBF by O-MF-BIA was 11.79% and by ADP was 12.00%, both in the "good" category per ACSM standards. Subjects were homogeneous in age and all identified as male. |
|  |  | (b) Indicate number of participants with missing data for each variable of interest | N/A | No missing data reported for any variables. |
|  |  | (c) *Cohort study*—Summarise follow-up time (eg, average and total amount) | N/A | Not applicable - cross-sectional study with single testing session. |
| Outcome data | 15* | *Cohort study*—Report numbers of outcome events or summary measures over time | *N/A* |  |
|  |  | *Case-control study—*Report numbers in each exposure category, or summary measures of exposure | *N/A* |  |
|  |  | *Cross-sectional study—*Report numbers of outcome events or summary measures | pp.4, Table 2 | Summary measures for all 54 participants reported: No statistical difference between O-MF-BIA and ADP for FFM (M=0.365 kg, p=.164), FM (M=-0.204 kg, p=.291), and PBF (M=-0.222%, p=.302). PBF measurements were 0.22% lower with BIA compared to ADP. Body composition comparison presented in Table 2. Limits of agreement: FFM (5.675 to -4.946), FM (5.092 to -5.499), PBF (5.899 to -6.343). Strong correlation for FFM (r(52)=.92, p<.001), FM (r(52)=.75, p<.001), PBF (r(52)=.70, p<.001). Explained variance: FFM 84%, FM 56%, PBF 49%. |
| Main results | 16 | (*a*) Give unadjusted estimates and, if applicable, confounder-adjusted estimates and their precision (eg, 95% confidence interval). Make clear which confounders were adjusted for and why they were included | pp.4, Table 2 | Summary measures for all 54 participants reported: No statistical difference between O-MF-BIA and ADP for FFM (M=0.365 kg, p=.164), FM (M=-0.204 kg, p=.291), and PBF (M=-0.222%, p=.302). PBF measurements were 0.22% lower with BIA compared to ADP. Body composition comparison presented in Table 2. Limits of agreement: FFM (5.675 to -4.946), FM (5.092 to -5.499), PBF (5.899 to -6.343). Strong correlation for FFM (r(52)=.92, p<.001), FM (r(52)=.75, p<.001), PBF (r(52)=.70, p<.001). Explained variance: FFM 84%, FM 56%, PBF 49%. |
|  |  | (*b*) Report category boundaries when continuous variables were categorized | p.4 | Limits of agreement boundaries provided: FFM (LOA 5.675 to -4.946), FM (LOA 5.092 to -5.499), PBF (LOA 5.899 to -6.343). BMI categorization referenced using WHO standards (25.9 kg/m² = "overweight"). PBF categorization referenced using ACSM standards (11.79-12.00% = "good" category). |
|  |  | (*c*) If relevant, consider translating estimates of relative risk into absolute risk for a meaningful time period | N/A | Not applicable - no risk estimates in this method comparison study. |

Continued on next page

| Other analyses | 17 | Report other analyses done—eg analyses of subgroups and interactions, and sensitivity analyses | N/A | No subgroup analyses, interaction analyses, or sensitivity analyses were conducted. Secondary analysis examined correlation between BMI and PBF: strong correlation with BIA (r(52)=.57, p<.001) and moderate correlation with ADP (r(52)=.41, p=.002). |
| --- | --- | --- | --- | --- |
| Discussion | | | | |
| Key results | 18 | Summarise key results with reference to study objectives | pp.4-5 | Primary finding: No significant differences between O-MF-BIA and ADP for measuring mean values of FFM, FM, and PBF in Division I male lacrosse athletes. Mean difference in PBF was 0.222%, which is well within the 5-6% acceptable range established by prior research. Strong correlations found between methods for FFM, FM, and PBF. However, wide limits of agreement noted between devices. Secondary finding: BMI was strongly to moderately associated with PBF as measured by BIA (r=.57) and ADP (r=.41), respectively. |
| Limitations | 19 | Discuss limitations of the study, taking into account sources of potential bias or imprecision. Discuss both direction and magnitude of any potential bias | pp.6-7 | Limitations explicitly discussed: (1) Hydration status was not measured, which can impact O-MF-BIA measurements; (2) Food intake prior to testing was not directly monitored despite fasting instructions; (3) Study included only male lacrosse athletes, limiting generalizability to other sexes or sports; (4) Wide limits of agreement despite lack of mean differences suggest devices should not be used interchangeably for individual assessments. Authors note the study was designed to imitate real-world athlete testing conditions where hydration monitoring is not typically feasible in strength and conditioning settings. |
| Interpretation | 20 | Give a cautious overall interpretation of results considering objectives, limitations, multiplicity of analyses, results from similar studies, and other relevant evidence | pp.6-7 | Cautious interpretation provided: While mean body composition measures were similar with moderate to strong correlations, the wide limits of agreement (FFM: 10.621 kg range, FM: 10.591 kg range, PBF: 12.242% range) suggest the two devices are not interchangeable. The authors recommend caution when comparing results derived from each device. O-MF-BIA may be a practical alternative to ADP in sports settings, but consistent use of the same device is recommended for long-term athlete monitoring. Results were compared extensively to previous literature with varying findings. Authors note devices may be suitable for population-level estimates but not for individual assessments when used interchangeably. |
| Generalisability | 21 | Discuss the generalisability (external validity) of the study results | p.7 | Generalizability explicitly addressed: "Because the current study included only male lacrosse athletes, the findings should not be generalized to other sexes or sports." The authors recommend that future research should expand to include other sports and examine the validity of O-MF-BIA for monitoring longitudinal body composition changes. |
| Other information | |  | | |
| Funding | 22 | Give the source of funding and the role of the funders for the present study and, if applicable, for the original study on which the present article is based | Pg. 7 | The authors state that no external funding was provided for the study. |

*Give information separately for cases and controls in case-control studies and, if applicable, for exposed and unexposed groups in cohort and cross-sectional studies.

**Note:** An Explanation and Elaboration article discusses each checklist item and gives methodological background and published examples of transparent reporting. The STROBE checklist is best used in conjunction with this article (freely available on the Web sites of PLoS Medicine at http://www.plosmedicine.org/, Annals of Internal Medicine at http://www.annals.org/, and Epidemiology at http://www.epidem.com/). Information on the STROBE Initiative is available at www.strobe-statement.org.
